# Spin-Coating and Aerosol Spray Pyrolysis Processed Zn_1−x_Mg_x_O Films for UV Detector Applications

**DOI:** 10.3390/nano12183209

**Published:** 2022-09-15

**Authors:** Vadim Morari, Veaceslav V. Ursaki, Emil V. Rusu, Victor V. Zalamai, Pascal Colpo, Ion M. Tiginyanu

**Affiliations:** 1 D. Ghitu Institute of Electronic Engineering and Nanotechnologies, 2028 Chisinau, Moldova; 2 National Center for Materials Study and Testing, Technical University of Moldova, 2004 Chisinau, Moldova; 3 Academy of Sciences of Moldova, 2001 Chisinau, Moldova; 4 European Commission, Joint Research Centre (JRC), Ispra, Italy

**Keywords:** thin ZnMgO films, spin coating, aerosol spray pyrolysis, crystalline phase structure, band gap, photodetector, current–voltage characteristics, injection photodiode

## Abstract

A series of Zn_1−x_Mg_x_O thin films with *x* ranging from 0 to 0.8 were prepared by spin coating and aerosol spray pyrolysis deposition on Si and quartz substrates. The morphology, composition, nano-crystalline structure, and optical and vibration properties of the prepared films were studied using scanning electron microscopy (SEM), energy dispersive X-ray analysis (EDX), X-ray diffraction (XRD), and optical and Raman scattering spectroscopy. The optimum conditions of the thermal treatment of samples prepared by spin coating were determined from the point of view of film crystallinity. The content of crystalline phases in films and values of the optical band gap of these phases were determined as a function of the chemical composition. We developed heterostructure photodetectors based on the prepared films and demonstrated their operation in the injection photodiode mode at forward biases. A device design based on two Zn_1−x_Mg_x_O thin films with different *x* values was proposed for extending the operational forward bias range and improving its responsivity, detectivity, and selectivity to UV radiation.

## 1. Introduction

Wurtzite-type zinc oxide is one of the most important multifunctional semiconductor compounds and is used in a wide range of applications, such as UV lasers and light-emitting diodes (LEDs) [1,2,3,4,5,6,7,8,9,10,11], photodetectors [12,13,14,15,16,17], gas and tactile sensors [18,19,20], solar cells [21,22,23], and transparent electronics [24], due to its unique physical properties, such as its wide bandgap of ~3.36 eV, large exciton binding energy of ~60 meV, high electron mobility, and high optical transparency. It also has a series of advantages over GaN-based materials, such as lower density of defects, stronger radiation hardness [14], and being an environmentally friendly material.

An important feature required for optoelectronic applications, e.g., in photodetectors and light emitting devices, is the possibility of controlling the material band gap for tuning the spectral range of emission or the optical sensitivity. One versatile way to achieve this function is alloying zinc oxide with Mg, since the band gap of ZnMgO solid solutions covers a wide ultraviolet (UV) spectral region ranging from the direct band gap of 3.36 eV in ZnO to that of 7.8 eV in MgO at room temperature.

ZnMgO films have been previously prepared by a variety of technological methods such as RF [12,15] and DC [25] magnetron sputtering, radio-frequency plasma-assisted molecular beam epitaxy (RF-MBE) [13,26,27], plasma-enhanced atomic layer deposition (PE-ALD) [28], pulsed laser deposition (PLD) [29,30], metal-organic chemical vapor deposition (MOCVD) [31], chemical bath deposition (CBD) [32], spray pyrolysis [27,33,34], and sol-gel spin coating [35,36,37]. CBD, spin coating, and spray pyrolysis are the most advantageous techniques from the point of view of processing cost, and they are suitable for the preparation of thin films applied in photodiodes.

The performances of photodiodes based on ZnMgO layers are strongly dependent not only on chemical compositions of layers, but also on their crystalline structure and texture. Since the wurtzite structure is a stable phase of ZnO, while MgO is stabilized in the rock salt structure, three types of textures are inherent to ZnMgO alloy films, namely, textures consisting of wurtzite-phase, cubic-phase, and mixed-phase crystallites. Respectively, ZnMgO photodetectors have been divided into three types depending upon the structures of alloys [14]. On the other hand, the p-n heterojunction photodetectors can be divided into two categories with respect to their operating mode, namely, classical photodiodes operating at reverse bias, and injection-mode devices working at forward bias [32,38,39,40]. The injection photodiodes operating at high forward bias voltage may have the advantage of higher integrated sensitivity as compared to diodes operating in the classical mode.

Most of ZnMgO photodetectors demonstrated to date have been based on films prepared by sputtering and MBE technologies, and partly by MOCVD growth, with the p-n heterojunction photodetectors being operated in the classical mode at reverse bias [14].

The goal of this paper is to investigate the properties of Zn_1−x_Mg_x_O films prepared by aerosol spray pyrolysis and spin coating deposition for the purpose of assessing their suitability for the development of p-n heterojunction photodetectors operating in the injection mode.

## 2. Sample Preparation and Experimental Details

Zn_1−x_Mg_x_O films with various *x*-values were deposited on quartz and p-Si substrates using two technological methods, namely, spin coating and aerosol spray pyrolysis. Solutions of zinc acetate dihydrate with 99.999% purity and magnesium acetate tetrahydrate with purity ≥99% purchased from Sigma-Aldrich (Sigma-Aldrich Chemie GmbH, Taufkirchen, Munich, Germany) were used in both approaches. 0.35 M zinc acetate and magnesium acetate solutions with various Mg/Zn ratios were dissolved in 20 mL of 2-methoxyethanol +0.5 mL of diethanolamine (DEA) for spin coating, or in ethanol for aerosol spray pyrolysis deposition. The prepared solutions were mixed for 30 min at the temperature of 50–60 °C in an ultrasonic bath before the deposition process.

Spin coating was performed at room temperature at a rotation speed of 3000 rpm in multiple coating cycles, with the rotation lasting 30 s for each cycle followed by drying the coated layer at 180 °C for 10 min. After the deposition of a certain number of layers, which determines the film thickness, the samples were treated at a temperature of 500 °C in air or in a mixture of air and argon (80:20%) for one hour.

The prepared solution was sprayed onto the substrate using a homemade sprayer with an O_2_ gas flow in the case of aerosol spray pyrolysis. The solution was injected into the oxygen gas flow by means of a syringe controlled by a stepper motor via a computer interface. The distance between the sprayer and the heated substrate was kept at 18 cm to produce a uniform coverage of the film on the substrate. The substrate temperature was 500 °C during the deposition process, the thickness of the film being determined by the duration of deposition and by the rate of precursor solution injection in the process.

The morphology investigation and chemical composition microanalysis of the produced films were performed by a FEI Helios Nanolab 600 Scanning Electron Microscope (SEM) equipped with an Ametek model ELECT PLUS X-ray detector (EDX) purchased from FEI Company (Field Electron and Ion Company, FEI), Hillsboro, OR, USA.

A Bruker AXS D8 DISCOVER X-ray diffractometer (Bruker Corporation, Billerica, MA, USA) with Cu Kα1 radiation (λ = 0.15406 nm) operated at 40 kV beam voltage and 40 mA beam current was used for X-ray diffraction measurements.

Raman scattering (RS) spectra were recorded at room temperature with a Renishaw InVia Qontor confocal microscope (Renishaw plc, Wotton-under-Edge, Stroud district of Gloucestershire, England, UK) equipped with a laser excitation source of 532 nm (50 mW). A (100×) microscope objective lens was selected to focus the light on the sample surface. The system calibration was performed on a monocrystalline Si wafer with a main peak measured at 521 cm^−1^. A total of 10 spectra were collected at 5 s exposure time and 5% laser power for each measurement.

Optical transmission spectra were measured at room temperature with a Jasco V-670 spectrometer (Jasco International CO., LTD., Tokyo, Japan).

Heterostructure photodetectors were prepared by deposition of one or two Zn_1−x_Mg_x_O thin films with different compositions on p-Si substrates, with subsequent deposition of the Al ohmic contact by thermal evaporation on the backside of the Si substrate, and the Ag front-side contact on the surface of the upper Zn_1−x_Mg_x_O film through a special mask, followed by sample annealing at the temperature of 300 °C in vacuum. The current–voltage characteristics and the photocurrent of the photodetector structures were measured using a Keithley 2400 Source Meter Unit (SMU) under radiation from a xenon DKSS-150 lamp passed through different optical filters.

## 3. Morphology, Composition, and Crystal Structure Characterizations of the Prepared Films

**Figure 1:**
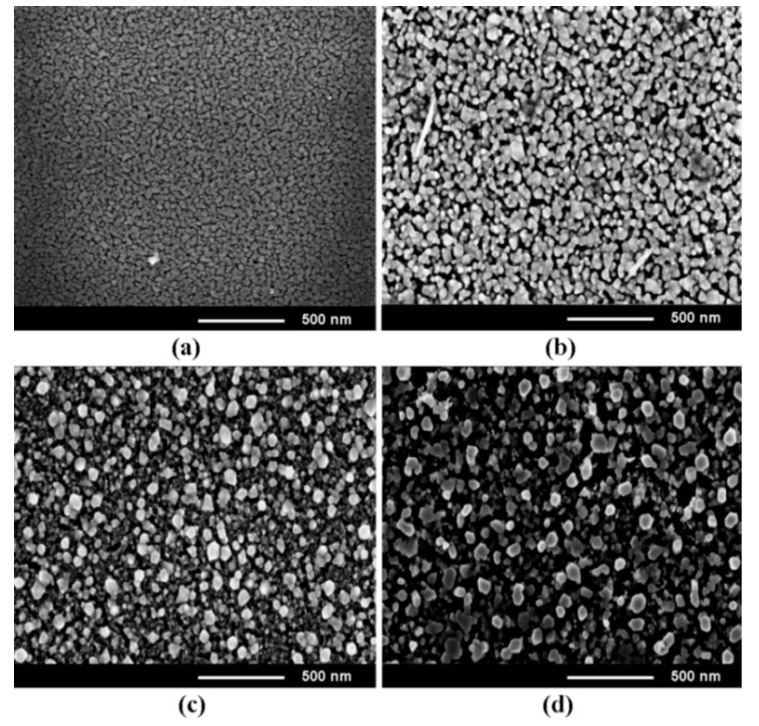
SEM images, top view of Zn_0.5_Mg_0.5_O films deposited by spin coating and annealed in air for 15 min (**a**), 30 min (**b**), 45 min (**c**), and 60 min (**d**).

Figure 1 shows the morphology of Zn_0.5_Mg_0.5_O films with thickness around 100 nm prepared by spin coating and annealed in air at 500 °C for various durations ranging from 15 to 60 min. One can see that the films consist of crystallites with basically a uniform distribution over the film surface, while the mean size of crystallites increases from around 30 nm to around 70 nm with increasing annealing time.

**Figure 2:**
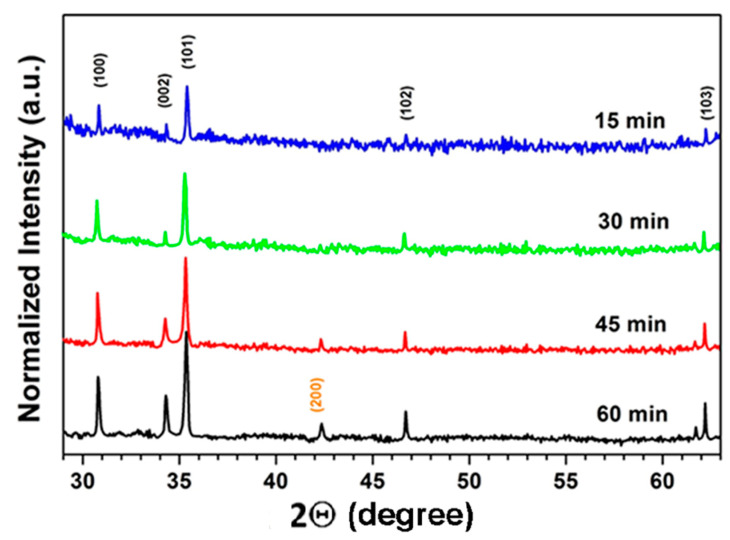
XRD pattern of Zn_0.5_Mg_0.5_O films deposited by spin coating and subjected to annealing in air at 500 °C for various durations.

The XRD analysis (Figure 2) suggests that concomitantly with increasing the crystallite sizes, the crystal quality of the deposited films improves with increasing the annealing time, as indicated by the enhancement of the intensity of XRD reflexes from the wurtzite phase indexed according to the PDF Card No. 01-078-3032. At the same time, the (200) reflex from the rock salt phase appears at annealing durations longer than 45 min.

The annealing temperature of 500 °C and the annealing duration of 1 h were found to constitute optimum conditions of thermal treatment, since the crystallite sizes and the crystal quality of the film improves with the increase of the annealing temperature up to 500 °C and the duration of annealing up to 1 h, as illustrated for the film with composition of Zn_0.5_Mg_0.5_O in Figure 1 and Figure 2. On the other hand, as previously reported [37], degradation of the film morphology and their cracking occurs with further increase of these technological parameters. Moreover, deviation from stoichiometry towards an excess of oxygen was observed at higher annealing temperatures and longer annealing durations, when the films were thermal treated in air. Similar trends have been observed for other film compositions.

**Figure 3:**
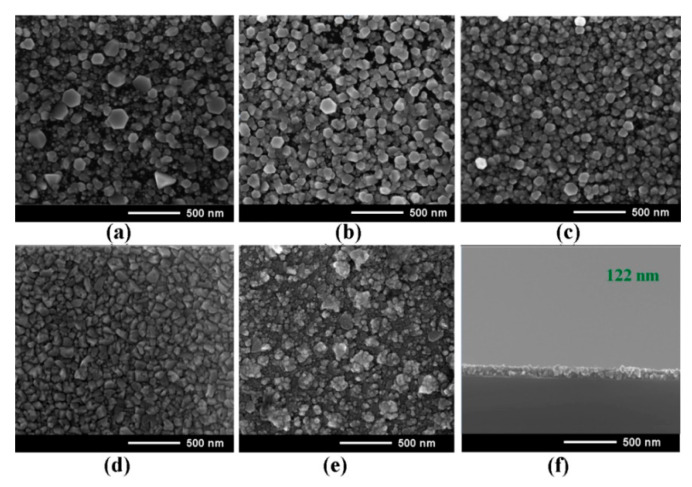
SEM images of Zn_1−x_Mg_x_O alloy films deposited by spin coating and subjected to annealing at 500 °C during 1 h. The *x*-values of the films: 0 (**a**); 0.2 (**b**); 0.4 (**c**); 0.6 (**d**); 0.8 (**e**). The SEM image in cross section of a film with *x* = 0.4 is shown in (**f**).

Figure 3 illustrates the evolution of the morphology of Zn_1−x_Mg_x_O films deposited by spin coating as a function of the Mg content in the films (*x* value). One can see that the crystallites with hexagonal shape are evidenced in films with low *x* value. Note that the crystallites become shapeless with the increase of the Mg content in the deposited films.

The results of the EDX analysis summarized in Table 1 suggest that the compositions of the Zn_1−x_Mg_x_O films are stoichiometric within the ±2% accuracy of the instrument, and they correspond to the *x*-values set in the zinc acetate–magnesium acetate solutions.

**Figure 4:**
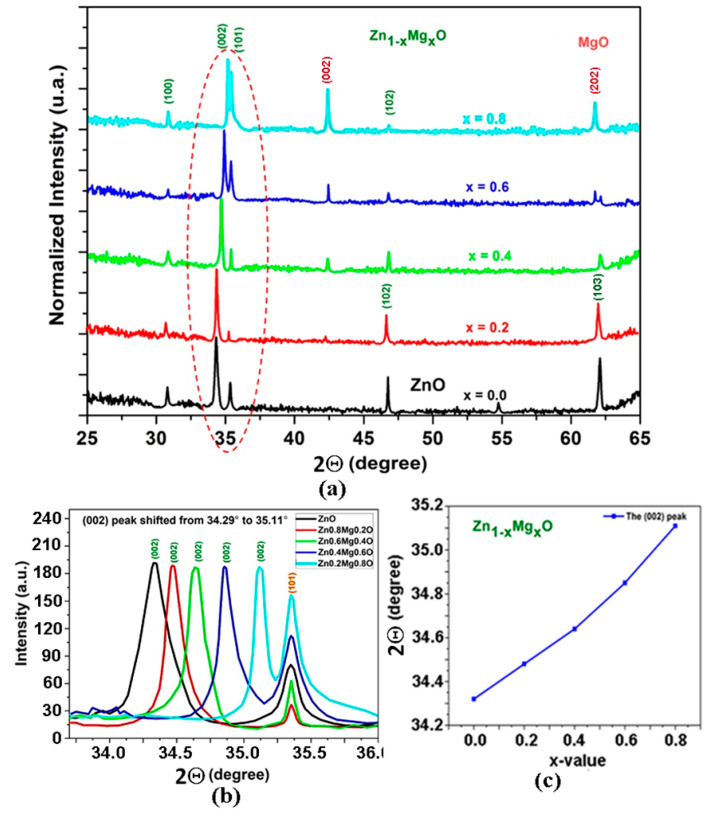
(**a**) XRD pattern of Zn_1−x_Mg_x_O films deposited by spin coating. (**b**) Evolution of the (002) reflex in the wurtzite phase with increase of the *x* value. (**c**) Dependence of the position of the (002) reflex on the *x* value.

The XRD pattern of the Zn_1−x_Mg_x_O films (0 < *x* < 0.8) prepared by spin coating is shown in Figure 4.

The XRD investigations suggest that the wurtzite phase is present in films up to the *x* value of 0.8. At the same time, (002) and (202) reflexes from the rock salt phase emerge in the pattern at *x* values higher than 0.4.

The gradual shift of the (002) reflex to larger refraction angles with increase of the *x* value suggests an efficient incorporation of Mg atoms into the wurtzite lattice. The position of this reflex approaches the position of a peak, which represents a superposition of the (101) reflex from the wurtzite phase and the (111) reflex from the rock salt phase.

**Figure 5:**
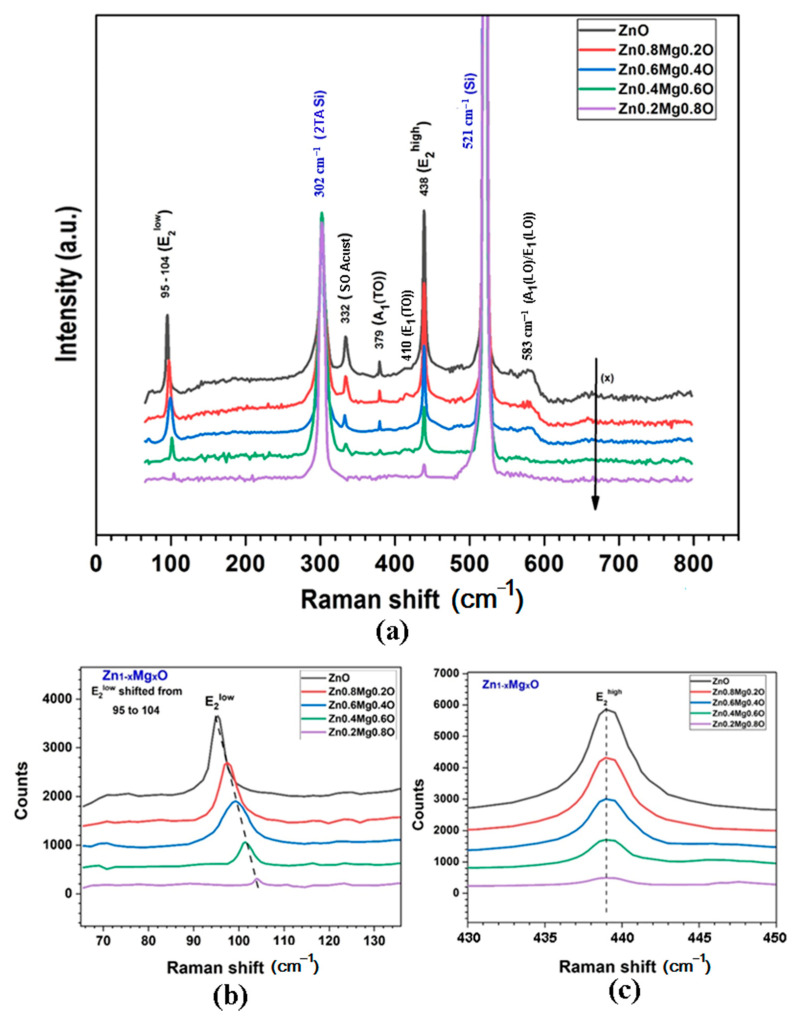
(**a**) RS spectra of Zn_1−x_Mg_x_O films deposited by spin coating. (**b**) Evolution of the E_2_^low^ mode in the wurtzite phase with increase of the *x* value. (**c**) Evolution of the E_2_^high^ mode with increase of the *x* value.

The XRD data are corroborated by the results of Raman scattering spectroscopy, which are indicative of efficient incorporation of the Mg atoms into the wurtzite lattice of Zn_1−x_Mg_x_O alloy films (Figure 5). Wurtzite ZnO belongs to the C_6v_ space group (P6_3_mc). According to group theory, the corresponding zone center optical phonons are of the following symmetry modes: A_1_ + 2B_1_ + E_1_ + 2E_2_ [41], of which the A_1_, E_1_, and 2E_2_ modes are the first-order Raman active, while the 2B_1_ phonons are silent. The A_1_ and E_1_ modes are split into LO and TO components.

All the Raman active phonon modes are clearly identified in the measured spectrum (Figure 5a). The peaks at around 100 cm^−1^ and 438 cm^−1^ are attributed to ZnO nonpolar optical phonon E_2_(low) and E_2_(high) modes, respectively. The peaks at 379 cm^−1^ and 410 cm^−1^ correspond to the A_1_(TO) and E_1_(TO) modes, respectively. The band at 583 cm^−1^ comes from a combination of A_1_(LO) and E_1_(LO) modes. Apart from that, the peak at 332 cm^−1^ is usually observed in the spectrum, it being attributed to second order Raman processes involving acoustic phonons [42].

The low frequency E_2_ mode is predominantly associated with the non-polar vibration of the heavier Zn sublattice, while the high frequency E_2_ mode predominantly involves the displacements of lighter oxygen atoms. Therefore, the incorporation of Mg atoms into the wurtzite lattice substituting the Zn atoms leads to shifting of the E_2_(low) mode to higher wavenumbers (Figure 5b), while the position of the E_2_(high) mode is basically stable (Figure 5c).

Furthermore, similar to the results of XRD analysis, the Raman spectra suggest that the wurtzite phase is present in films up to the *x* value of 0.8, since the E_2_(low) and E_2_(high) modes persist in the spectrum. At the same time, the intensity of RS modes coming from the wurtzite phase gradually decreases with increase of the *x* value, indicating the formation of the rock salt Raman inactive phase.

**Figure 6:**
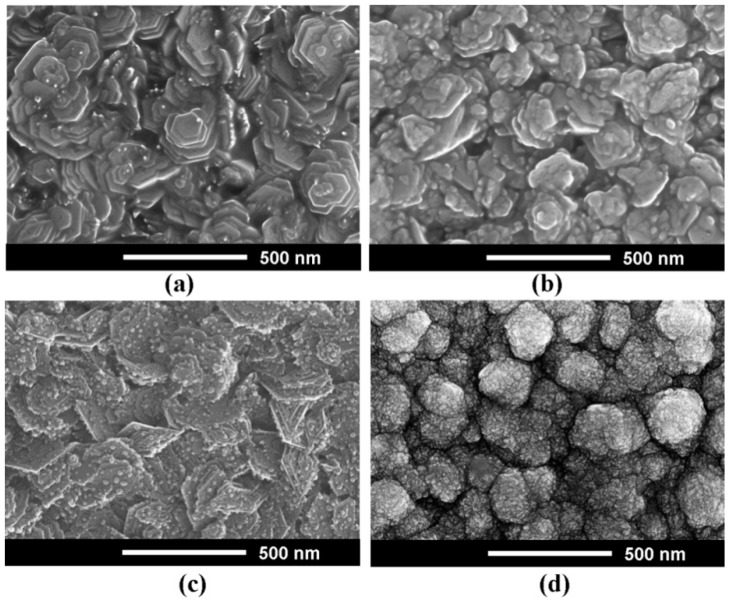
SEM images (top view) of Zn_1−x_Mg_x_O alloy films deposited by aerosol spray pyrolysis method with the *x* values of 0.2 (**a**); 0.4 (**b**); 0.6 (**c**); and 0.8 (**d**).

The morphology of Zn_1−x_Mg_x_O alloy films prepared by aerosol spray pyrolysis also changes with the increase of the Mg content in films, similarly to that of the films prepared by spin coating (Figure 6). Crystallites with hexagonal shape are also present in films prepared by aerosol spray pyrolysis with low *x* value. However, their shape is more complex, with hexagonal platelets deposited on each other, therefore forming some kind of flower. Similar to films prepared by spin coating, the shape of these structures deteriorates with the increase of the *x* value, which results in the formation of shapeless crystallites.

The results of the EDX analysis summarized in Table 2 suggest that the compositions of the Zn_1−x_Mg_x_O films prepared by aerosol spray pyrolysis are stoichiometric within the ±2% accuracy of the instrument, and they correspond to the *x*-values set in the zinc acetate-magnesium acetate spray solutions.

**Figure 7:**
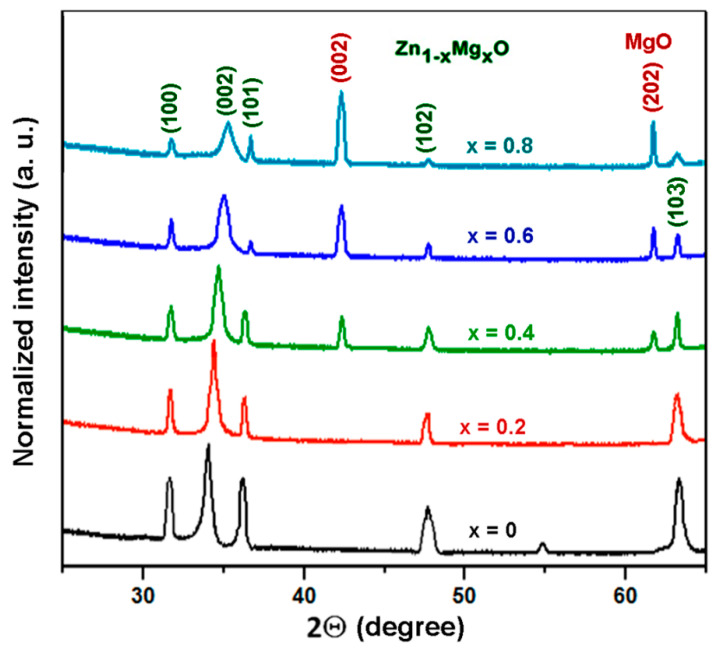
XRD pattern of Zn_1−x_Mg_x_O alloy films deposited by aerosol spray pyrolysis.

The XRD data for Zn_1−x_Mg_x_O alloy films prepared by aerosol spray pyrolysis reveal the same trends as those observed in films deposited by spin coating (Figure 7).

The (002) reflex is gradually shifted to larger diffraction angles when increasing the *x* value. The intensity of reflexes coming from the wurtzite phase decreases as the *x* value increases higher than 0.4, with a concomitant emerging of (002) and (202) reflexes from the cubic rock salt phase. Nevertheless, reflexes from the wurtzite phase persist in the pattern registered for the *x* value of 0.8, suggesting that the wurtzite phase is present in films up to an *x* value of 0.8.

**Figure 8:**
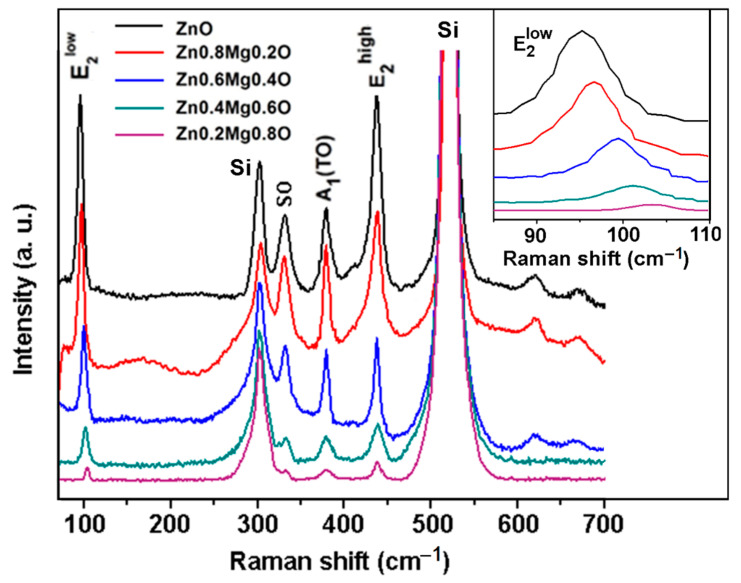
RS spectra of Zn_1−x_Mg_x_O films deposited by aerosol spray pyrolysis. Evolution of the E_2_^low^ mode with increasing *x* value is shown in the inset.

Similar to samples prepared by spin coating, the XRD data collected on samples prepared by aerosol spray pyrolysis are corroborated by the results of RS spectroscopy presented in Figure 8. The intensity of Raman modes coming from the wurtzite phase decreases with the increase of the *x* value in Zn_1−x_Mg_x_O films, and the E_2_^low^ mode shifts to higher wavenumbers.

## 4. Optical Properties and Band Gap Analysis of the Prepared Films

**Figure 9:**
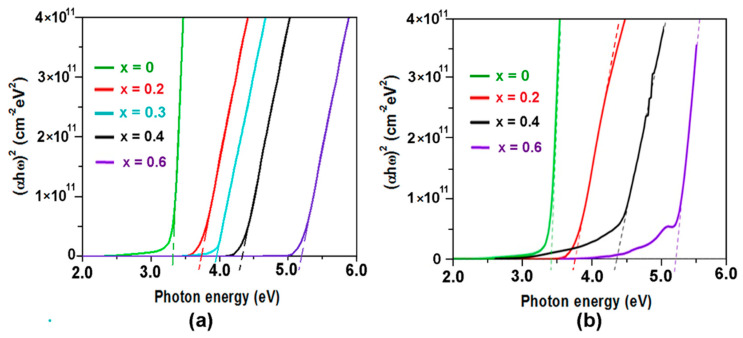
Tauc plot of optical absorption spectra measured at room temperature for Zn_1−x_Mg_x_O films deposited on quartz substrates by spin coating (**a**) and aerosol spray pyrolysis (**b**).

The Zn_1−x_Mg_x_O alloy films prepared by both the spin coating and aerosol spray pyrolysis methods proved to be highly transparent, with a transparency level greater than 80% in the visible spectral range up to 3.3 eV. In accordance with the Tauc formula [43,44], the optical band gap of the films was determined from the point of intersection of the linear segment of the plot presented in Figure 9 with the photon energy axis. The deviation of curves from the linear dependence at lower value of the absorption coefficient can be explained taking into account the local compositional fluctuations and rock salt phase inclusions in the wurtzite phase, leading to the formation of deep band tails in the band gap, as discussed in a previous paper [37].

**Figure 10:**
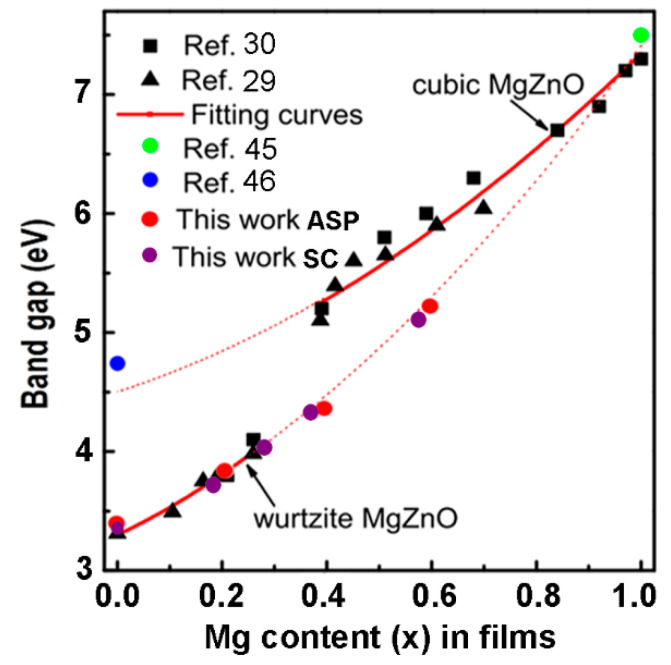
Dependence of the band gap of Zn_1−x_ Mg_x_O films on the Mg content *x* in films deposited by spin coating (SC) and by aerosol spray pyrolysis (ASP).

The optical band gap deduced from the Tauc plot is presented in Figure 10 as a function of the Mg content in the films (*x*). The obtained data are compared with previously reported data in the literature for films prepared by PLD [29,30,45,46]. The experimental data are also fitted to the standard bowing equation corresponding to the rock salt phase (upper curve) and to the wurtzite phase (lower curve).

According to [30], the band gap is fitted to the wurtzite phase up to the *x* value of 0.27, and to the cubic phase down to the *x* value of 0.4. On the other hand, the data of the present work show that the band gap of the films prepared by both the spin coating and the spray pyrolysis methods are fitted by the curve corresponding to the wurtzite phase up to the *x* value of 0.6. These data suggest that, in spite of the fact that the films represent a mixture of wurtzite and cubic phases at *x* values higher than 0.4, as indicated by the XRD analysis, the wurtzite phase is a predominant one and it determines the optical band gap of films with *x* value up to 0.6. This observation hints to the possibility of extending the band gap of the wurtzite-type ZnMgO films grown by spin coating or aerosol spray pyrolysis towards the solar-blind-region. Up to now, a solar-blind 4.55 eV band gap of a wurtzite type Zn_1−x_Mg_x_O film with the *x* value of 0.55 was achieved by RF-plasma assisted MBE growth using quasi-homo buffers on a suitable substrate [47,48,49].

## 5. Heterostructure Photodetectors Characterization

**Figure 11:**
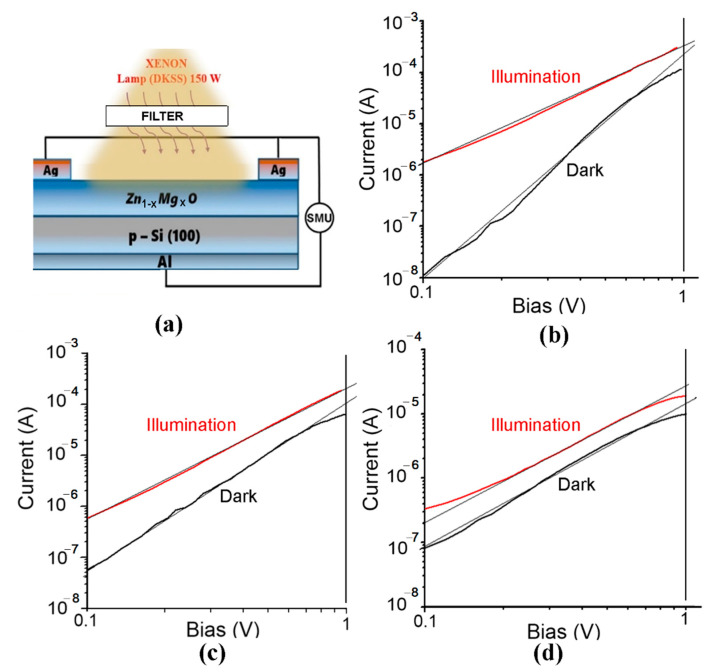
Design of n-Zn_1−x_Mg_x_O/p-Si heterostructure photodiodes (**a**) and current–voltage characteristics of devices, fabricated on films deposited by spin coating, plotted for forward bias in a double logarithmic scale for *x* values in films of 0.1 (**b**), 0.2 (**c**), and 0.4 (**d**).

The design of n-Zn_1−x_Mg_x_O/p-Si heterostructure photodiodes is shown in Figure 11a, while the current–voltage characteristics of devices plotted for forward bias in a double logarithmic scale for films with the *x* value of 0.1, 0.2, and 0.4 are shown in Figure 11b–d, respectively. The thickness of Zn_1−x_Mg_x_O layers was around 120 nm, as shown in Figure 3f. The characteristics have been measured under UV illumination centered at 300 nm with optical power of 63 mW focused on a spot with area of 0.125 cm^2^, resulting in a power density of around 500 mW⋅cm^−2^.

Note that the current–voltage characteristics plotted with linear voltage axis and logarithmic voltage axis do not fit the classical formula for a p-n junction, $I=I_{S}\left[\exp\left(\frac{qU}{nkT}\right)-1\right]$, where *I*_S_ is the saturation current, *q* is the elementary charge, *k* is the Boltzmann constant, *T* is the temperature, and *n* is the ideality factor of a classical diode [37]. The plot should be a straight line in such semi-logarithmic coordinates. However, this is not the case for the prepared diode structures. On the contrary, the characteristics fit a straight line in double-logarithmic coordinates, as deduced from Figure 11b–d, which means that it corresponds to a power function I ∝ U*^n^*, according to the Lampert theory [50].

The *n* value under illumination is about 2, which corresponds to the space charge limited (SCL) current injection according to the Mott–Gurney (MG) law [51,52]. The fact that the investigated heterojunctions work at forward bias with MG law characteristics while classical diodes work at reverse bias suggests that the prepared heterojunctions work as injection photodiodes. Similar devices have been previously demonstrated on Si-CdS [40], CdS-CdTe [53], and CdS-CdSTe-ZnCdTe [39] heterostructures.

The *n* value for the dark current characteristics in Figure 11 decreases from around 4 in Zn_1−x_Mg_x_O films with *x* value of 0.1 to around 3 for the *x* value of 0.2, and to around 2 for the *x* value of 0.4. The ratio of the photocurrent to the dark current at the bias of 1 V is around 3 for all the *x* values, while at the bias of 0.1 V, this ratio decreases from around 100 for the *x* value of 0.1 to around 10 for the *x* value of 0.2, and to around 3 for the *x* value of 0.4. However, the responsivity (R) is quite low at the bias of 0.1 V for all the values of *x*, being around (10–30) µA⋅W^−1^. The responsivity is higher at the bias of 1V, but it decreases from around 3 mA⋅W^−1^ for the *x* value of 0.1 to around 2 mA⋅W^−1^ for the *x* value of 0.2, and around 0.1 mA⋅W^−1^ for the *x* value of 0.4.

**Figure 12:**
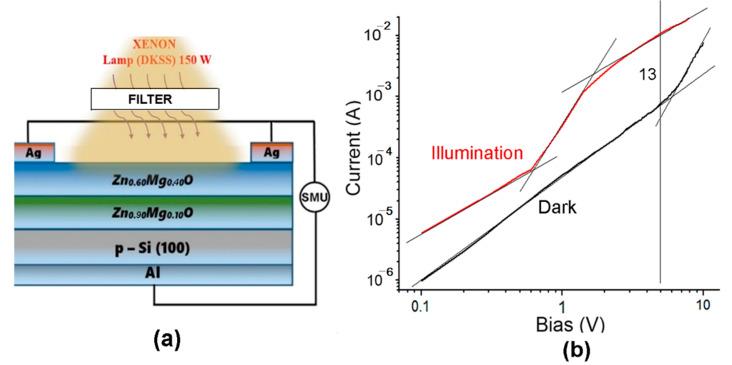
(**a**) Design of an Al/p-Si/n-Zn_90_Mg_10_O/n-Zn_60_Mg_40_O/Ag photodiode. (**b**) Current–voltage characteristics of the device fabricated on films deposited by spin coating plotted for the forward bias in a double logarithmic scale.

The parameters of these devices are overall low because the ratio of the photocurrent to the dark current decreases with increasing bias voltage, and the operation of devices at voltages higher than 1 V becomes ineffective. A device structure with two Zn_1−x_Mg_x_O films with different *x* values was proposed to overcome this drawback, as shown in Figure 12a. A more complex band diagram is inherent to such a device, and the Zn_0.60_Mg_0.40_O upper layer with a wider band gap plays the role of absorption layer, which protects the base layer with the composition of Zn_0.90_Mg_0.10_O and is expected to reduce the density of surface states.

The current–voltage characteristic of the photodiode fits a straight line in the double logarithmic coordinates at bias voltages up to 5 V in the dark with a power function index *n* of around 2, while the characteristic under illumination has a more complex shape, with three segments fitting a straight line. The second segment in the voltage range of (0.7–2) V has a power function index *n* value higher than 3, leading to the increase of the ratio of the photocurrent to the dark current at biases higher than 1 V. The ratio I_photo_/I_dark_ reaches a value of 13 at the voltage of 5 V, which improves the parameters of the device.

The n-Zn_90_Mg_10_O film plays the role of device base. Since the diode works at forward bias and the main portion of the bias is applied on this film, the p-n junction injects minority charge carriers through the direct current flow. An equivalent number of the majority carriers flow to the semiconductor from the upper contact via the Zn_60_Mg_40_O film to compensate for the space charge in the Zn_90_Mg_10_O film. When increasing the injection current, the concentration of the non-equilibrium carriers significantly exceeds the concentration of equilibrium ones, and the non-equilibrium carriers determine the conductivity of the base region. The applied bias is distributed between the p-n junction and the base region. Since the resistance of the base region decreases as a result of injection, the portion of the bias voltage on the p-n junction increases, therefore leading to the increase of the injection in the base and to a further decrease of its resistance. A similar redistribution of the applied bias voltage occurs under illumination. Therefore, an effect of intrinsic injection amplification of the primary photocurrent occurs. On the other hand, the generation of photo-carriers occurs in the upper Zn_60_Mg_40_O film, which has a significant impact on the current–voltage characteristic under illumination, which results in an increase of the power function index *n* at voltages higher than 0.7 V, thus improving the I_photo_/I_dark_ ratio as compared to devices with single Zn_1−x_Mg_x_O films. Apart from that, the upper film improves the selectivity of the device to UV radiation.

**Figure 13:**
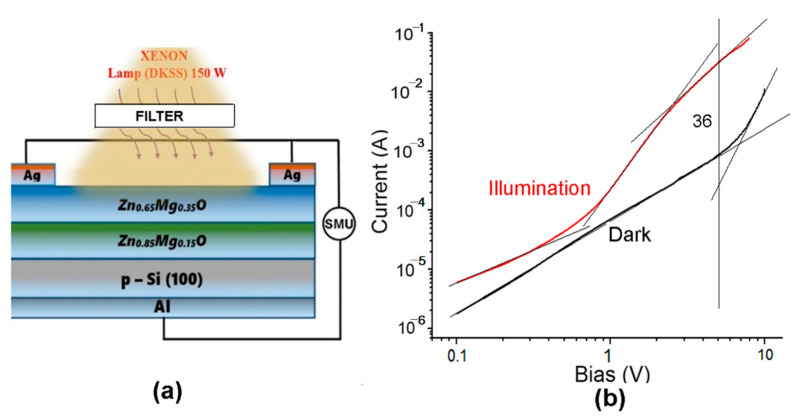
(**a**) Design of an Al/p-Si/n-Zn_85_Mg_15_O/n-Zn_65_Mg_35_O/Ag photodiode. (**b**) Current–voltage characteristics of the device fabricated on films deposited by aerosol spray pyrolysis plotted for the forward bias in a double logarithmic scale.

The current–voltage characteristics are even better for a device with the *x* value of 0.35 instead of 0.40 in the upper Zn_1−x_Mg_x_O film and the *x* value of 0.15 instead of 0.10 in the lower film (Figure 13). The ratio I_photo_/I_dark_ reaches a value of 36 at the voltage of 5 V for such a device structure. The energy band diagram of the p-Si/n-Zn_85_Mg_15_O/n-Zn_65_Mg_35_O heterojunction has been discussed in a previous paper [33].

The parameters of the photodetectors (responsivity and detectivity) were calculated from the experimental data according to Formulas (1) and (2), respectively [54]:

(1) $$R=\frac{I_{photo}-I_{dark}}{P_{ill}},$$

(2) $$D^{*}=\frac{R\sqrt{A}}{\sqrt{2eI_{dark}}}$$

where *I_photo_* is the current under illumination, *I_dark_* is the dark current, *P_ill_* is the illumination power (63 mW), *A* is the active area of the photodetector (0.125 cm^2^) and *e* is the elementary charge. The parameters of the prepared photodetectors are summarized in Table 3.

**Figure 14:**
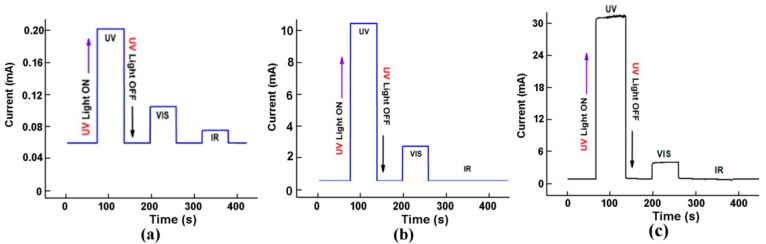
Relaxation of photocurrent measured at 300 K with illumination at different wavelengths for a photodetector with a Zn_80_Mg_20_O film (**a**), a photodetector with n-Zn_90_Mg_10_O/n-Zn_60_Mg_40_O films (**b**), and a photodetector with n-Zn_85_Mg_15_O /n-Zn_65_Mg_35_O films (**c**).

The design with two Zn_1−x_Mg_x_O films also improves the selectivity of devices, as illustrated in (Figure 14). Such devices are practically not sensitive to infrared (IR) radiation, in contrast to devices with a single Zn_1−x_Mg_x_O film. The device based on n-Zn_85_Mg_15_O/n-Zn_65_Mg_35_O films is less sensitive to visible radiation as compared to UV radiation by a factor of 9, while this ratio is 4.5 for the device with the n-Zn_90_Mg_10_O/n-Zn_60_Mg_40_O films and is around 3 for the device with a single Zn_80_Mg_20_O film.

## 6. Conclusions

The results of this study demonstrate the preparation of Zn_1−x_Mg_x_O thin films by spin coating and aerosol spray pyrolysis on Si substrates with homogeneous morphology over the entire surface of the films. The optimum temperature of thermal treatment of the films prepared by spin coating from the point of view of crystallinity is 500 °C for 1 h. The nano-crystallites become shapeless with increasing Mg content in films. According to the results of XRD analysis, the films prepared by both methods are of wurtzite structure up to an Mg content of around 40%, as demonstrated by the results of XRD analysis. However, the wurtzite phase is present in films up to an Mg content of 80%, forming a composite with the cubic rock salt phase. The efficient incorporation of Mg atoms into the wurtzite lattice is demonstrated by the results of XRD and Raman scattering analysis.

The results of optical measurements demonstrate that the optical bandgap of films prepared by both methods is determined by the wurtzite phase up to the *x* value of 0.60, in spite of the fact that the films represent a mixture of wurtzite and cubic phases at *x* values higher than 0.4. This observation hints at the possibility of extending the band gap of the wurtzite-type ZnMgO films grown by spin coating or aerosol spray pyrolysis towards the solar-blind-region.

The analysis of the current–voltage characteristics of photodetectors developed on the basis of the prepared films suggests their operation in the injection photodiode mode at forward biases. A device design with two Zn_1−x_Mg_x_O thin films with different *x* values was proposed for extending the operational forward bias range. Such a design proved to significantly improve the responsivity and detectivity, as well as the selectivity of the injection photodiodes to UV radiation. In spite of different morphologies of the films prepared by spin coating and aerosol spray pyrolysis, the photodiodes on demonstrate similar characteristics when fabricated on films with the same thickness.

## Figures and Tables

**Table 1:** Chemical composition, according to the EDX-analysis, of the Zn_1−x_Mg_x_O alloy films deposited by spin coating.

*x*-Value	Element	Weight %	Atomic %
0	O	22.38	51.66
	Zn	77.62	48.34
	Mg	0	0
0.2	O	24.49	50.11
	Zn	66.96	39.24
	Mg	8.56	10.65
0.4	O	25.29	50.70
	Zn	59.47	29.19
	Mg	15.24	20.11
0.6	O	30.56	49.78
	Zn	41.70	19.29
	Mg	27.74	30.93
0.8	O	33.12	48.84
	Zn	26.75	09.58
	Mg	40.13	41.58

**Table 2:** Chemical composition, according to the EDX-analysis, of the Zn_1−x_Mg_x_O alloy films deposited by the aerosol spray pyrolysis.

*x*-Value	Element	Weight %	Atomic %
0	O	21.19	51.35
	Zn	78.81	48.65
	Mg	0	0
0.2	O	22.57	50.67
	Zn	70.52	39.08
	Mg	6.91	10.25
0.4	O	24.33	49.45
	Zn	60.28	29.98
	Mg	15.39	20.57
0.6	O	27.74	49.47
	Zn	49.49	30.25
	Mg	22.77	20.28
0.8	O	31.62	49.13
	Zn	28.25	10.49
	Mg	40.13	40.38

**Table 3:** Parameters of photodetectors on the basis of Zn_1−x_Mg_x_O films.

Photodetector Structure Design	Responsivity (R), mA⋅W^−1^	Detectivity (D*), cm⋅Hz^1/2^⋅W^−1^
Zn_0.9_Mg_0.1_O/Si	3.0	2.0 × 10^8^
Zn_0.8_Mg_0.2_O/Si	2.2	1.8 × 10^8^
Zn_0.6_Mg_0.4_O/Si	0.1	2.0 × 10^7^
Zn_0.9_Mg_0.1_O/Zn_0.6_Mg_0.4_O/Si	150	3.5 × 10^9^
Zn_0.85_Mg_0.15_O/Zn_0.65_Mg_0.35_O/Si	460	1.0 × 10^10^

## Data Availability

The data presented in this study are available on request from the corresponding authors.
